# Carbapenem-resistant and carbapenem-susceptible isogenic isolates of *Klebsiella pneumoniae* ST101 causing infection in a tertiary hospital

**DOI:** 10.1186/s12866-015-0510-9

**Published:** 2015-09-03

**Authors:** Meritxell Cubero, Guillermo Cuervo, M. Ángeles Dominguez, Fe Tubau, Sara Martí, Elena Sevillano, Lucía Gallego, Josefina Ayats, Carmen Peña, Miquel Pujol, Josefina Liñares, Carmen Ardanuy

**Affiliations:** Department of Microbiology, Hospital Universitari de Bellvitge, University of Barcelona-IDIBELL, Barcelona, Spain; Centro de Investigacion Biomédica en Red de Enfermedades Respiratorias (CIBERES), Instituto de Salud Carlos III, Madrid, Spain; Department of Infectious Diseases, Hospital Universitari de Bellvitge, University of Barcelona-IDIBELL, Barcelona, Spain; Spanish Network for Research in Infectious Diseases (RD06/0008), Instituto de Salud Carlos III, Madrid, Spain; Department of Immunology, Microbiology, and Parasitology, Faculty of Medicine and Dentistry, University of the Basque Country (UPV/EHU), Bizkaia, Spain

## Abstract

**Background:**

In this study we describe the clinical and molecular characteristics of an outbreak due to carbapenem-resistant *Klebsiella pneumoniae* (CR-KP) producing CTX-M-15 and OXA-48 carbapenemase. Isogenic strains, carbapenem-susceptible *K. pneumoniae* (CS-KP) producing CTX-M-15, were also involved in the outbreak.

**Results:**

From October 2010 to December 2012 a total of 62 CR-KP and 23 CS-KP were isolated from clinical samples of 42 patients (22 had resistant isolates, 14 had susceptible isolates, and 6 had both CR and CS isolates). All patients had underlying diseases and 17 of them (14 patients with CR-KP and 3 with CS-KP) had received carbapenems previously. The range of carbapenem MICs for total isolates were: imipenem: 2 to >32 μg/ml vs. <2 μg/ml; meropenem: 4 to >32 μg/ml vs. <2 μg/ml; and ertapenem: 8 to >32 μg/ml vs. <2 μg/ml. All the isolates were also resistant to gentamicin, ciprofloxacin, and cotrimoxazole. Both types of isolates shared a common PFGE pattern associated with the multilocus sequence type 101 (ST101). The *bla*_CTX-M-15_ gene was detected in all the isolates, whereas the *bla*_OXA-48_ gene was only detected in CR-KP isolates on a 70 kb plasmid.

**Conclusions:**

The clonal spread of *K. pneumoniae* ST101 expressing the OXA-48 and CTX-M-15 beta-lactamases was the cause of an outbreak of CR-KP infections. CTX-M-15-producing isolates lacking the *bla*_OXA-48_ gene coexisted during the outbreak.

## Background

Nosocomial outbreaks due to multidrug-resistant enterobacteria have become a serious problem worldwide, especially due to the spread of extended spectrum beta-lactamase (ESBL)-producing strains and to the posterior emergence of carbapenemases [1]. In Europe, carbapenem resistance among *K. pneumoniae* isolates is related to the presence of multidrug-resistant isolates producing beta-lactamases belonging to the KPC, VIM, or OXA-48 families, making the treatment of infections difficult due to these microorganisms [1].

In Spain, carbapenem-resistant *K. pneumoniae* (CR-KP) strains have been described sporadically due to production of VIM-type enzymes and, more recently, OXA-48 [2, 3]. In addition, after whole genome sequencing of a ST101 OXA-48-producing *K. pneumoniae*, revealed that the *bla*_OXA-48_ gene was recently found in IncL/M plasmids previously described [4, 5].

In our hospital, a tertiary teaching hospital in Spain, an active surveillance program for ESBL- and carbapenemase-producing enterobacteria has been ongoing since the 1990s [6]. In this study we describe the clinical and molecular epidemiology of a nosocomial outbreak due to a *K. pneumoniae* clone producing CTX-M-15 and OXA-48. Interestingly, isogenic *K. pneumoniae* isolates producing the CTX-M-15 enzyme but lacking the *bla*_OXA-48_ gene were also detected during the same period of time.

## Methods

### Ethics statement

This study was approved by the Clinical Research Ethics Committee of Bellvitge University Hospital (PR334/14). Written informed consent was not necessary for this retrospective study as it was part of our standard microbiological routine. Patient data were anonymized for the purposes of this analysis, and all confidential patient information was protected in accordance with Spanish law.

### Hospital setting

The study was carried out in the Hospital Universitari de Bellvitge, a 900-bed university teaching hospital for adult patients in Barcelona (Spain) that provides medical and surgical care in all areas except pediatrics, obstetrics, and burns. The hospital serves an area of 600,000 inhabitants and admits an average of 24,000 patients per year.

### Bacterial isolates and clinical data collection

This retrospective study was initially designed to identify CR-KP isolates, although the results obtained drew our attention to the importance of CS-KP present during the same period. Computerized clinical charts of patients with CR-KP or their clonally related CS-KP isolates were retrospectively reviewed in order to collect clinically relevant data: demographics, clinical features, treatments, and outcomes. From October 2010 to December 2012, all CR-KP and CS-KP isolates were selected from clinical samples.

The clinical relevance assessment was determined in accordance with the CDC/NHSN surveillance definition of health care-associated infection in patients with samples from any body site positive for CR-KP, but without related signs or symptoms of infection. In those cases, patients were considered to be colonized [7].

### Antimicrobial susceptibility testing and molecular typing

Antimicrobial susceptibility was tested by microdilution using the commercial method MicroScan® (Siemens, USA).

The presence of carbapenemases was tested by the double-disk synergy test between disks containing carbapenems and disks with amoxicillin/clavulanic acid, EDTA, and boronic acid.

The MICs of different antimicrobials were interpreted using the new EUCAST breakpoints valid from 2015 (http://www.eucast.org/clinical_breakpoints/): imipenem (IPM): susceptible, MIC ≤ 2 μg/ml and resistant, MIC > 8 μg/ml; meropenem (MEM): susceptible, MIC ≤ 2 μg/ml and resistant, MIC > 8 μg/ml; ertapenem: susceptible, MIC ≤ 0.5 μg/ml and resistant, MIC > 1 μg/ml; gentamicin (GEN): susceptible, MIC ≤ 2 μg/ml and resistant, MIC > 4 μg/ml; amikacin (AMK): susceptible MIC ≤8 μg/ml and resistant, MIC > 16 μg/ml; trimethoprim-sulfamethoxazole (SXT): susceptible, MIC ≤ 2 μg/ml and resistant, MIC > 4 μg/ml; and ciprofloxacin (CIP): susceptible, MIC ≤ 0.5 μg/ml and resistant, MIC > 1 μg/ml.

The genetic relatedness of all multidrug-resistant *K. pneumoniae* isolates was tested by pulsed field gel electrophoresis (PFGE) of chromosomal DNA after restriction with *Xba*I*.* Band patterns were analyzed by visual inspection following the criteria described by Tenover et al. [8]. Strains differing in 3 or less bands were considered subtypes. Isolates from the different PFGE subtypes were selected for multi-locus sequence typing (MLST). MLST was performed as described on the MLST website of the Institute Pasteur: http://bigsdb.web.pasteur.fr/klebsiella/klebsiella.html. The allele number and sequence type (ST) were assigned using this MLST website.

### Characterization of the multidrug-resistance pattern

#### Beta-lactamase characterization

A multiplex PCR assay described by Fang et al., which includes detection of *bla*_OXA_, *bla*_CTX-M_, *bla*_TEM_, and *bla*_SHV,_ was used for beta-lactamase detection in all *K. pneumoniae* isolates [9]. For CTX-M enzymes a second PCR was performed to discriminate between *bla*_CTX-M-9_ group, *bla*_CTX-M-10_ group, and *bla*_CTX-M-1_ group [10]. Other carbapenemase genes were detected by PCR amplification following previously published procedures: class A carbapenemases (KPC, IMI, NMC, GES) [11], metallo-beta-lactamases (IMP-1, IMP-2, VIM-1, VIM-2, GIM, SIM, SPM) [11], and the oxacillinase (OXA-48) [12]. PCR products were purified and sequenced in both strands using the amplification primers at Macrogen Inc. (Korea).

### Plasmid analysis

Plasmid analysis and location of the genetic support of the OXA-48 carbapenemase were performed. Plasmid DNA from two CR-KP and two CS-KP clinical isolates was extracted using the GeneJET plasmid miniprep kit (Thermo Scientific), and was analyzed by electrophoresis on 0.7 % agarose gels. Plasmid size was determined by comparison with those from the standard strains *Escherichia coli* NCTC 50193 (CECT678) and NCTC 59192 (CECT679), ranging in size from 163.3 to 2 kb. The *K. pneumoniae* 7680 strain, containing a plasmid with the *bla*_OXA-48_ gene, was used as a positive control. *Escherichia coli* strains CECT678 and CECT679 were used as molecular weight markers (http://www.straininfo.net). CECT678 contains natural plasmids of 54.38 kb, 7.30 kb, 5.56 kb, 5.14 kb, 3.98 kb, 3.08 kb, 2.71 kb, and 2.06 kb; CECT679 contains plasmids of 154 kb, 66.2 kb, 37.6 kb, and 7.4 kb [13]. To locate the *bla*_OXA-48_ gene, gels with plasmid DNA were transferred to a nylon membrane by the Southern technique and hybridized with a PCR-generated OXA-48 probe, labeled with dUTP-digoxigenin. Detection of hybrids was done using an antidigoxigenin antibody coupled to alkaline phosphatase following the manufacturer’s indications (Roche Diagnostics).

## Results

### Outbreak description

**Fig. 1:**
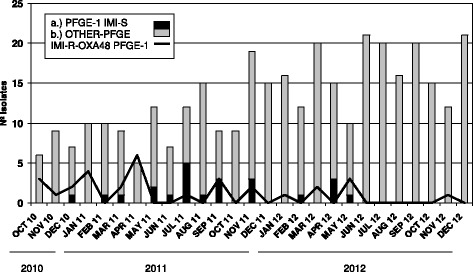
Detection of clinical samples with ESBL-producing *K. pneumoniae* and non-ESBL-producing *K. pneumoniae*. Black bars represent CS-KP ESBL strains belonging to the PFGE-1 pattern and gray bars represent non-PFGE-1 pattern ESBL *K. pneumoniae*. Black line represents patients with CR-KP non-ESBL strains harboring the *bla*_OXA-48_ gene that belonged to the PFGE-1 pattern

The first isolation of the carbapenem-resistant *Klebsiella pneumoniae* (CR-KP) occurred in late October 2010, in a patient who developed a surgical site infection (intra-abdominal abscess) due to a complication during surgery for colon cancer. A total of 62 CR-KP isolates were obtained from 28 hospitalized patients from October 2010 to December 2012. The outbreak was initially restricted to the Digestive Surgery Unit but it was subsequently spread throughout the Intensive Care Unit (ICU). A total of 42.85 % of infected patients were detected in the period January-April 2011 (maximum peak of incidence) (Fig. 1).

During a routine control of ESBL-producing *K. pneumoniae* by molecular typing (PFGE) we also detected some carbapenem-susceptible *K. pneumoniae* (CS-KP) isolates, all of them with the same PFGE pattern as the CR-KP epidemic strain (denominated PFGE-1). The ESBL-CS-KP strains were isolated from February 2011 to May 2012 in 23 clinical samples from 14 patients, whereas the non-ESBL-CS-KP isolates were mainly detected between July and November 2011 (50 % of the isolates identified in 7 patients). However, their carbapenem-resistant counterparts were present until the end of the study period. Thereafter, a gradual decrease in new cases of CR-KP with the same PFGE pattern was detected in relation to general control measures implemented during the third trimester of 2011.

Throughout the whole period, six patients had CR-KP and CS-KP isolates in different clinical samples (*n* = 26 isolates). In four of these patients the resistant CR-KP isolates preceded the susceptible CS-KP one. In Fig. 1 a single isolate is represented for each patient; patients with both CR-KP and CS-KP isolates are represented just once as presenting the CR-KP isolate.

**Fig. 2:**
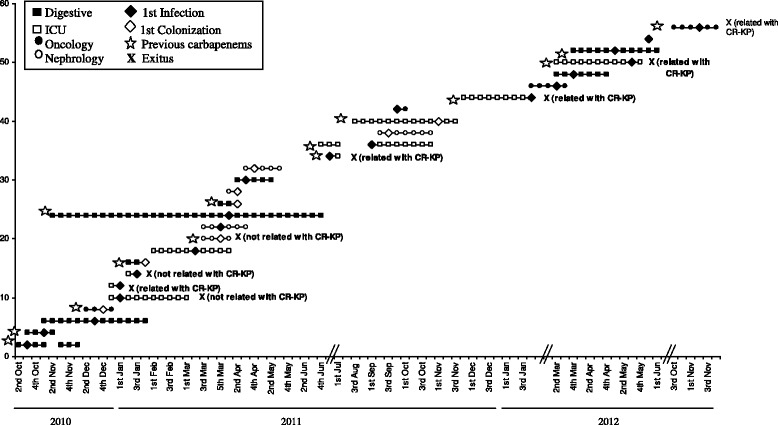
Clinical features of the 28 patients with carbapenem-resistant *K. pneumoniae* isolates. Each line represents one patient from his/her hospital admission until discharge or death. Squares and circles only represent time units (one week) in hospital with no samples obtained on those days. Previous carbapenem treatment is marked with a star drawn on the left of each patient line. The moment of infection or colonization is indicated with a diamond shape

The clinical timelines for the 28 patients with isolation of CR-KP (22 CR-KP and 6 with both isolate types) are shown in Fig. 2. Fourteen of the patients with CR-KP isolates had previously received carbapenems (marked with a star). Most patients with CR-KP isolates were admitted to ICU and surgical wards.

### Clinical characteristics, therapy, and outcomes

Table 1 shows the clinical characteristics and demographics of patients with clinical samples yielding CR-KP or CS-KP. Patients with both strains (CR- and CS-KP) were analyzed only in the group of CR-KP isolates. Overall, there were no differences in terms of demographics, site of isolation, or comorbid conditions.

**Table 1:** Epidemiological and clinical data of patients with CR-KP and CS-KP isolates

	CR-KP	CS-KP	p-value
	*N* = 28	*N* = 14	
Mean age (SD)	62.93 (+/- 14.491)	69.62 (+/- 16.717)	0.186
Male sex	18 (64.3 %)	7 (50 %)	0.508
Site of isolation			
Urine	10 (35.7 %)	9 (64.3 %)	0.107
Blood culture	6 (21.4 %)	2 (14.3 %)	0.697
Surgical wound	4 (14.3 %)	1 (7.1 %)	0.650
Abdominal samples	3 (10.7 %)	0	0.539
Respiratory samples	4 (14.3 %)	1 (7.1 %)	0.643
Others	1 (3.6 %)	1 (7.1 %)	1
Length of stay before acquisition (SD)	28.43 (+/- 31.319)	20.14 (+/- 31.705)	0.430
Overall mortality (related CR- or CS-KP)^a^	35 % [7/20]	11.11 % [1/9]	0.371 (0.280)
	(20 % [4/20])	(0)	
ICU acquisition	9 (32.1 %)	0	0.019*
Previous antibiotic therapy (treatment)			
Penicillins/Betalactamase Inhibitor	8 (28.6 %)	2 (14.3 %)	0.451
Carbapenems	14 (50 %)	3 (21.4 %)	0.102
Third generation cephalosporins/Monobactams	2 (7.1 %)	1 (7.1 %)	1
Fluoroquinolones	4 (14.3 %)	3 (21.4 %)	0.668
Trimethoprim-sulfamethoxazole	0	2 (14.3 %)	0.106
None	0	3 (21.4 %)	0.032*
Treatment			
Penicillins/Betalactamase Inhibitor	1 (3.6 %)	0	1
Carbapenems	3 (10.7 %)	7 (50 %)	0.008*
Carbapenems + Amikacin	1 (3.6 %)	1 (7.1 %)	1
Tigecycline	6 (21.4 %)	1 (7.1 %)	0.392
Tigecycline + Amikacin	1 (3.6 %)	0	1
Aminoglycosides	1 (3.6 %)	0	1
Colistin	4 (14.3 %)	0	0.283
Fosfomycin	1 (3.6 %)	0	1
None	1 (3.6 %)	0	1

*p < 0.05 being considered significant

^a^Patients with non-significant isolation of CR-KP or CS-KP, considered colonized, were excluded from the mortality analysis

Prior antibiotic therapy was more frequent among patients with CR-KP (28/28 vs. 11/14; *p* = 0.032) and especially involved carbapenems, although the difference in this latter respect did not reach statistical significance (14/28 vs. 3/14; *p* = 0.102).

Twenty out of 28 patients with clinical CR-KP samples were considered to have an infection due to this microorganism. Of these, 19 received antibiotic treatment (as well as other adjunctive therapies in some cases), which in 17 cases was considered appropriate according to the susceptibility test: tigecycline (*n* = 7, one of them in combination with AMK), carbapenems other than ertapenem (*n* = 4, in which cases the bacteria exhibited an intermediate sensitivity to the carbapenem selected, one of them in combination with AMK), colistin (*n* = 4), AMK (*n* = 1), and fosfomycin (FOF) (*n* = 1). Twelve of these 17 patients (70.58 %) recovered after treatment, while the other five died (three of them probably related to the CR-KP infection). The remaining three patients did not receive appropriate antimicrobial therapy: one patient treated with amoxicillin/clavulanic acid (AMC) survived, another treated with piperacillin/tazobactam (TZP) died due to a septic shock related to CR-KP, and the third did not receive treatment and died.

Nine of 14 patients with clinical samples yielding CS-KP were considered infected. Of these, eight were treated with carbapenems (one of them in combination with AMK) and one with tigecycline. Only one patient treated with IPM died (due to a urinary sepsis), although CS-KP did not appear to be the primary cause of death.

There was a significant difference in the use of carbapenems as antibiotic treatment among infected patients (3/20 vs. 7/9; *p* = 0.002). No other statistical difference was detected among CR-KP and CS-KP patients.

### Phenotypic and molecular characterization of the isolates

By disk diffusion all CR-KP isolates showed synergy between AMC and carbapenems, and the Hodge test was positive, suggesting carbapenemase activity. By contrast, CS-KP isolates did not present these phenotypic characteristics. CR- and CS-KP isolates were multidrug resistant. Table 2 shows the range of MICs of different antimicrobial agents. IPM MICs of CR-KP isolates ranged from 2 to >32 μg/ml, MEM MICs from 4 to >32 μg/ml, and ertapenem MICs from 8 to >32 μg/ml. All isolates with MICs of IPM >32 μg/ml were resistant to cefoxitin (FOX) (MIC >16 μg/ml). Using current EUCAST breakpoints, all the isolates had ertapenem MICs in the resistant range (breakpoint for resistance MIC >1 μg/ml).

**Table 2:** Characteristics of CR-KP and CS-KP isolates

	CR-KP	CS-KP
Number of different patients	28	14
Period of isolation	Oct 2010- Dec 2012	Dec 2010- May 2012
Betalactamase production	CTX-M-15 and OXA-48	CTX-M-15
PFGE profile and subtypes	PFGE-1	PFGE-1 and 6 subtypes
MLST	101	101
	Antimicrobial susceptibility	
Antimicrobial	MIC range (mg/L)	MIC range (mg/L)
Gentamycin	>8	>8
Amikacin	<8	<8
Cotrimoxazole	>4	>4
Ciprofloxacin	>2	>2
Imipenem	2 / >32	<2
Meropenem	4 / >32	<2
Ertapenem	8 / >32	<8

A common PFGE pattern was observed in all CR-KP and CS-KP isolates that was different to those observed in other ESBL-producing *K. pneumoniae* isolates collected in the same period (data not shown). Among CR-KP isolates there was a single PFGE pattern type designated PFGE-1, whereas CS-KP isolates showed the same PFGE-1 pattern with six different subtypes (Table 2). Ten representative isolates from the different subtypes (4 CR-KP and 6 CS-KP isolates) were typed by MLST and showed an identical allelic profile: *gap*A (allele number 2), *inf*B (allele number 6), *mdh* (allele number 1), *pgi* (allele number 5), *pho*E (allele number 4), *rpo*B (allele number 1), and *ton*B (allele number 6), associated with ST101.

The *bla*_CTX-M_ gene was detected by PCR in all CR-KP and CS-KP isolates, whereas the *bla*_OXA-48_ gene was only present in CR-KP isolates. After sequencing, the beta-lactamases were identified as CTX-M-15 and OXA-48. The remaining carbapenemase genes were not detected by PCR.

### Plasmid analysis

**Fig. 3:**
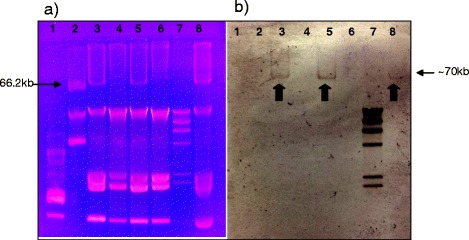
Plasmid profile of CR-KP and CS-KP isolates. **a** Agarose gel electrophoresis of *K. pneumoniae* plasmid DNA, and **b** Southern blot hybridization using a *bla*_OXA-48_ specific probe. Arrows show the presence of a ~70 kb OXA-48-containing plasmid in the CR-KP samples. Lane 1: CECT 678 (54.38 kb, 7.30 kb, 5.56 kb, 5.14 kb, 3.98 kb, 3.08 kb, 2.71 kb, and 2.06 kb plasmids); Lane 2: CECT 679 (154 kb, 66.2 kb, 37.6 kb, and 7.4 kb plasmids); and Lane 7: Low range ladder as controls. Lanes 3 and 5: CR-KP clinical isolates. Lanes 4 and 6: CS-KP clinical isolates. Lane 8: Positive control *K. pneumoniae* strain (*K. pneumoniae* 7680)

Southern blot hybridization analysis of plasmid DNA from two resistant and two susceptible clinical isolates identified a plasmid of approximately 70 kb in the CR-KP isolates. This plasmid had the same size as the control strain (*K. pneumoniae* 7680). Southern blot hybridization with the OXA-48 probe demonstrated the presence of the *bla*_OXA-48_ gene within this plasmid (Fig. 3). Plasmid analysis of representative carbapenem resistant and carbapenem susceptible clinical strains (2 of each) revealed the presence of 6 and 5 plasmids respectively, ranging in size from 1.9 Kb to 70 kb. Plasmid profile was identical in both CS-KP and CR-KP strains except for the presence of an extra 70 kb structure in the CR-KP isolates.

## Discussion

In the present study the carbapenem MICs of the CR-KP isolates producing OXA-48 had a broad range. Following the EUCAST criteria, CR-KP isolates had carbapenem MICs in the intermediate or resistant range, with the exception of seven isolates which had IPM MICs of 2 μg/ml. In addition, all OXA-48-producing isolates had ertapenem MICs ranging from 4 to >32 μg/ml, which is considered resistant. It is known that ertapenem MICs are easily altered in carbapenemase-producing isolates, and this is reflected in our observations here. Therefore, ertapenem resistance could be a good microbiological alert for detecting isolates with the presence of these enzymes. It should be noted that isolates with higher carbapenems MICs (for instance, IPM >32 μg/ml) were also FOX-resistant, suggesting that other mechanisms, such as the loss of porins could be involved in these resistant strains, as previously described [2, 11]. Other authors have suggested the efficacy of temocillin for OXA-48 producers screening and quickly recognition. In our experience (data not shown), although both isolates were temocilin-resistant, OXA-48 producers had no inhibition zone while a small zone was observed in the other strains [14]. Therapeutic options in patients infected by CR-KP are very limited. Indeed, beta-lactam resistance mediated by OXA-family beta-lactamases is known to be poorly inhibited by beta-lactamase inhibitors [15]. Therefore, and based on our experience, combinations of a beta-lactam plus beta-lactamase inhibitor should not be used for the treatment of CR-KP-producing OXA-48 enzymes. Other beta-lactam antibiotics, such as aztreonam and expanded spectrum cephalosporins, are theoretically useful in infections caused by OXA-48-producing clones, although the association of a CTX-M-15 in this outbreak precluded their use in our patients [16].

According to recent reports, there is limited evidence to support the use of tigecycline, which appears to show low effectiveness for treating CR-KP infections [15]. In our experience this antibiotic was effective (5 of 7 patients cured), although in three cases a surgical drainage of the abscess was also needed. Two patients treated with tigecycline died, although in one case the death was not related to the infection, while the other death was due to a bacteremic septic shock. Therapeutic options in patients infected by CR-KP are, therefore, very limited, and clinicians must often resort to old antibiotics with a non-negligible toxicity, as is the case of colistin, which was successfully used in four patients in our series [16, 17].

The use of carbapenems to treat OXA-48-producing enterobacteria with MICs of carbapenems in the intermediate range is a controversial issue. In recent outbreaks, sporadic cases of reported treatment have not shown good results [18], although the volume of information is very limited. In the present study, four patients with CR-KP infections received carbapenems (cases in which the bacteria exhibited an intermediate sensitivity to the carbapenem selected), and in two of these cases the carbapenem was associated with another active antibiotic (AMK or FOF). In this scenario the inoculum effect is probably an important variable that could explain the favorable outcome of our patients treated with carbapenems [16], in whom the antibiotic treatment supplemented the extraction of catheters and/or the drainage of collections, adjunctive therapies which undoubtedly meant a drastic reduction of the inoculum [19]. The probable usefulness of continuous infusion regimens was not tested in any of our patients [20].

Carbapenem resistance in *K. pneumoniae* is a growing problem in the hospital setting that limits the use of this antibiotic group for therapeutic purposes. There are several families of enzymes that are responsible for this increase in resistance, mainly KPC and VIM [1]. However, an increase in resistance to carbapenems due to OXA-48 carbapenemase-producing clones has recently been observed, especially associated with nosocomial outbreaks in Mediterranean countries, the first of which occurred in Turkey in 2004 [12].

This resistance determinant has now been detected worldwide in several bacterial species such as *Escherichia coli, Enterobacter cloacae* [21], and *Citrobacter freundii* [4], although the predominant species are *E. coli* and *K. pneumoniae*. In the present study we documented a clonal outbreak due to *K. pneumoniae* producing CTX-M-15 and OXA-48 carbapenemase. The sequence type of the epidemic strain (ST101) producing both enzymes (CTX-M-15 and OXA-48) has also been detected in other European and Mediterranean countries such as Israel and Tunisia [2, 4, 22], suggesting a continental spread of this multidrug-resistant lineage that has not yet been eradicated [23]. The dissemination across Spain and Europe of the OXA-48 enzyme has been described in several studies [2, 24, 25]. The most prevalent *K. pneumoniae* clones are ST101, ST11, and ST405, although in our hospital we have only detected OXA-48 isolates with ST101. The OXA-48 enzyme has been detected in various well-described clones of *K. pneumoniae*: ST101, ST11, ST14, ST15, ST147, ST395, and ST405 have already been reported in European and Mediterranean countries [23–28].

Patients affected by CR-KP isolates in our hospital shared certain characteristics with those of other outbreaks in our geographic area [2, 24, 25]. They were patients with significant comorbidities who had been exposed to major antibiotic pressure and whose infection was hospital-acquired. These infections were related to previous surgery or other invasive procedures (central venous catheters or urinary catheters). Fourteen patients received carbapenems prior to the CR-KP isolation. Although no case control studies have been published, previous carbapenem therapy has been identified in many studies describing CR-KP outbreaks [29]. In fact, this risk factor was more frequently identified in patients with CR-KP isolates than in those with CS-KP isolates.

The coproduction of ESBL with the *bla*_OXA-48_ gene is frequent and mainly related to the presence of plasmids [5]. In our study the co-existence of carbapenem-resistant and susceptible strains could be explained by the presence of the *bla*_OXA-48_ and *bla*_CTX-M-15_ genes in independent elements. The transmissibility of the plasmid-encoded *bla*_OXA-48_ favored the rapid spread of these strains, so the acquisition of *bla*_OXA-48_ by the carbapenem-susceptible strain could have occurred in our institution. The *bla*_OXA-48_ gene was detected located on a 62-kb IncL/M plasmid or on a 70-kb plasmid, indicating that a plasmid of variable size was mainly responsible for the spread of this gene [4, 21].

## Conclusions

Multi-resistant isolates of European carbapenem-resistant OXA-48 *K. pneumoniae* clone (ST101), previously identified in other Spanish hospitals and harboring plasmids carrying the *bla*_OXA-48_ gene, have been producing an outbreak in our hospital in Barcelona (Spain). In conclusion, the European carbapenem-resistant OXA-48 *K. pneumoniae* clone (ST101) is spreading among Spanish hospitals. Previous carbapenem use was identified as a common factor in affected patients. These multidrug-resistant isolates were successfully treated with tigecycline, colistin, or carbapenems combined with other antibiotics and adjunctive therapies. The spread of these strains suggests a plausible scenario in which the acquisition of new resistance determinants may make a strain extremely drug-resistant. Thus, these results highlight the need for surveillance of all multidrug-resistant *K. pneumoniae* isolates, not only those that produce carbapenemase or ESBL.
